# Navigating the Diagnosis of Atypical Still’s Disease in the Absence of the Characteristic Rash

**DOI:** 10.7759/cureus.106812

**Published:** 2026-04-10

**Authors:** David Villanueva-Lechuga, Alejandra Servin-Martinez, Luis Fernando Muñoz Chavez, Leonor Bonilla Quezada, Amanda Carreon Simental, Rene Castaneda-Flores, Luis Montiel López

**Affiliations:** 1 Internal Medicine, National Autonomous University of Mexico, Mexico City, MEX; 2 Internal Medicine, Centro Médico Nacional 20 de Noviembre, Mexico City, MEX; 3 Internal Medicine, Instituto de Seguridad y Servicios Sociales de los Trabajadores del Estado (ISSSTE), Mexico City, MEX

**Keywords:** adult-onset still’s disease, atypical still’s disease, autoinflammatory diseases, fever of unknown origin, yamaguchi criteria

## Abstract

Although adult-onset Still’s disease (AOSD) typically presents with a triad of fever, rash, and arthritis, atypical manifestations can significantly hinder the diagnostic process of fever of unknown origin (FUO). We describe the case of a 57-year-old woman who presented with prolonged fever, serositis, and extreme hyperferritinemia (ferritin levels 117,904 ng/mL), further complicated by aseptic encephalitis. Following a comprehensive diagnostic workup to exclude infectious, neoplastic, and other autoimmune etiologies, the patient was evaluated using the Yamaguchi criteria. Despite the absence of the characteristic salmon-pink rash, the diagnosis of atypical AOSD was confirmed as the patient fulfilled three major (fever, arthralgia, and neutrophilic leukocytosis) and two minor (lymphadenopathy and/or splenomegaly and the absence of rheumatoid factor and antinuclear antibodies) criteria. Clinical remission and resolution of neurological symptoms were achieved through treatment with systemic corticosteroids and tocilizumab (8 mg/kg). This case emphasizes that AOSD remains a critical differential diagnosis in FUO even without an evanescent rash and highlights the potential for severe neurological involvement. Our findings support the utility of the Yamaguchi criteria in atypical presentations and illustrate the rapid therapeutic response to interleukin-6 inhibition in this clinical context.

## Introduction

Fever of unknown origin (FUO) is clinically defined as a documented body temperature exceeding 38.3 °C on at least two occasions, persisting for a minimum of three weeks, without a definitive diagnosis after three days of appropriate inpatient or three outpatient evaluation visits [1]. To optimize diagnostic strategies, FUO is traditionally classified into four categories based on clinical context: classic, nosocomial, immunodeficiency-related, and travel-associated FUO [1]. Despite advances in diagnostic tools, approximately 8% of FUO cases remain undiagnosed [2].

Adult-onset Still's disease (AOSD) is a rare, systemic autoinflammatory disorder of unknown etiology characterized by a heterogeneous clinical presentation [3]. The classic triad includes high fever with spikes, evanescent rash, and inflammatory joint involvement [4]. Other manifestations may include odynophagia, myalgia, lymphadenopathy, splenomegaly, and liver dysfunction with elevated liver enzymes [4]. Neurological complications in AOSD are rare but well-documented, with aseptic encephalitis representing an uncommon manifestation within this already infrequent category. The largest single-center study found that 7.5% of AOSD inpatients (14 of 187 patients) developed neurological involvement, with aseptic meningitis being the most common presentation in 64.3% of neurological cases, while encephalitis was listed among "other rare manifestations" alongside cranial nerve palsy and cerebral infarction [5]. Aseptic encephalitis in AOSD appears predominantly as case reports in the literature, underscoring its rarity [6].

Laboratory abnormalities typically include marked neutrophilic leukocytosis and extreme hyperferritinemia. Since none of these findings is pathognomonic, AOSD remains a diagnosis of exclusion among the causes of fever of unknown origin [4].

Clinical diagnosis is guided by the Yamaguchi criteria, which require fulfillment of at least five criteria, including a minimum of two major criteria [3,6]. Although a characteristic salmon-pink macular or maculopapular rash is observed in approximately 75% of patients, its absence, while uncommon, does not exclude the diagnosis [7]. Fautrel’s criteria (published in 2002) are often considered more specific than those of Yamaguchi, as they incorporate modern biological markers such as glycosylated ferritin [8,9]. Unlike the Yamaguchi criteria, Fautrel’s classification assigns specific diagnostic weight to ferritin levels. In AOSD, total ferritin is typically massively elevated, while the glycosylated fraction drops below 20%. This finding is highly suggestive of AOSD and aids in differentiating it from other inflammatory or infectious diseases, where ferritin levels may rise, but the glycosylated fraction remains within normal limits. However, glycosylated ferritin testing was not available at our center [7].

## Case presentation

A 57-year-old woman with no relevant past medical history presented with bilateral swelling of the dorsum of the hands and feet, accompanied by arthralgia of the proximal interphalangeal joints that worsened with movement. Subsequently, she developed daily intermittent fever with evening predominance, reaching up to 39°C, associated with night sweats, chills, and headache. She reported an 8 kg weight loss over two months, as well as palpitations accompanied by resting tachycardia of up to 120 beats per minute. She also presented with spontaneous bruising on the forearm on two occasions, each lasting approximately three days and resolving without intervention. Additional symptoms included asthenia, adynamia, dyspnea on moderate exertion, and non-productive, non-cyanotic cough, prompting medical evaluation and hospital admission for workup of fever of unknown origin.

On initial assessment, she was tachycardic, with the remainder of her vital signs within normal limits. She exhibited dyspnea on minimal exertion, edema, and chills. Physical examination revealed cervical lymphadenopathy, bilateral pleural effusion, a palpable mass in the right breast, and palpable hepatomegaly. Mammography and breast ultrasound were performed, followed by a breast biopsy, which was negative for malignancy. Laboratory studies showed leukocytosis of 20,000 cells/mm³, with neutrophilia, moderate eosinophilia (2,000 cells/mm³), monocytosis, and marked hyperferritinemia with levels up to 117,904 ng/mL (normal 11-307 ng/mL).

Thoracoabdominal computed tomography demonstrated bilateral pleural effusions with associated atelectasis, as well as lymph node conglomerates in the left axillary region and cervical levels IV and V, predominantly on the left side, along with hepatosplenomegaly.

**Figure 1:**
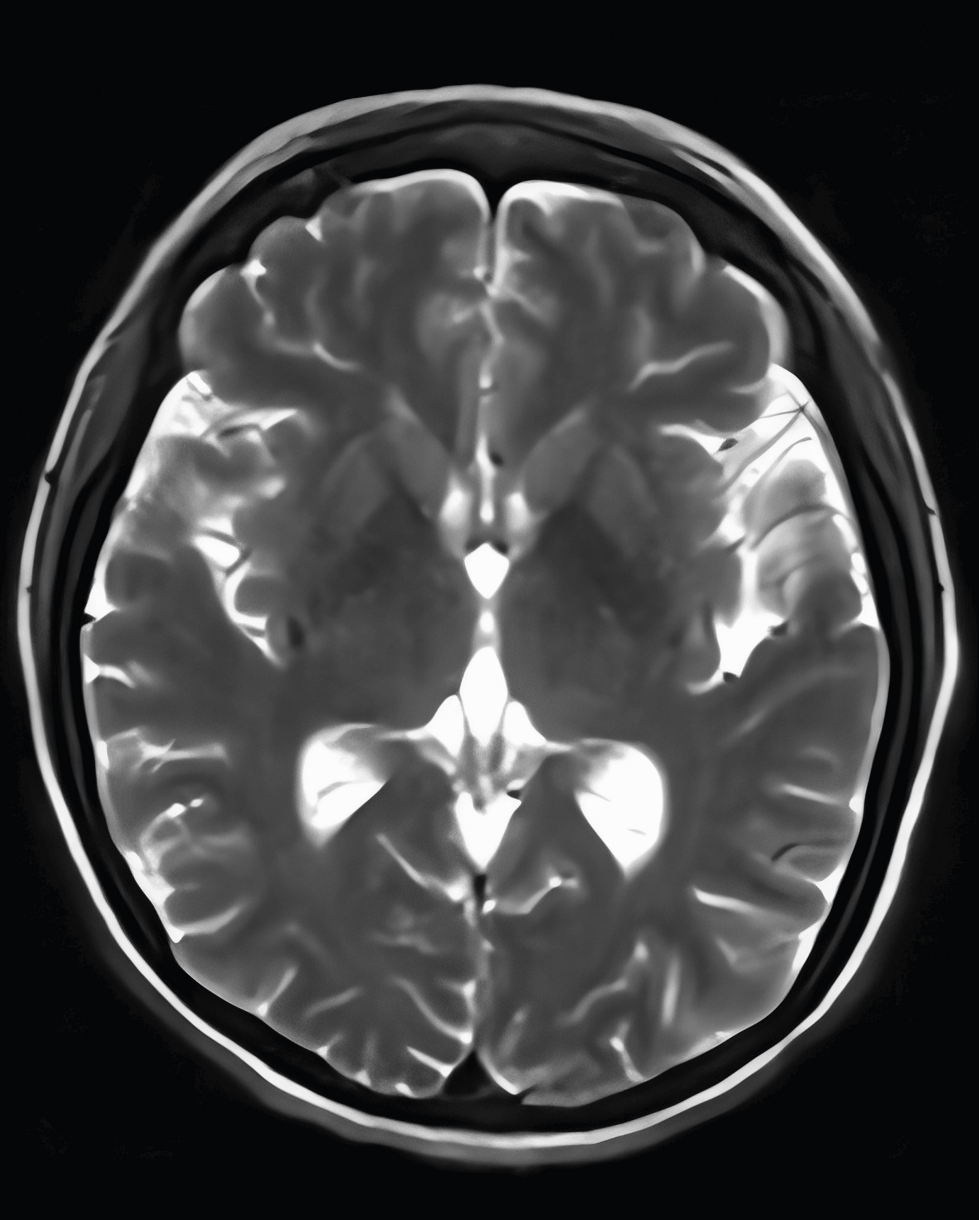
Brain MRI Imaging revealed heterogeneous diploic marrow with a discrete diffuse hyperintense area toward the bilateral frontoparietal convexity on T2 and FLAIR sequences, associated with cortical hyperintensity and diffusion-related T2 shine-through in the left frontoparietal, right frontal, and left temporo-occipital regions, alongside leptomeningeal enhancement of the left cerebral hemisphere and right temporo-occipital region, and continuous dural enhancement with supratentorial thickening.

During hospitalization, the patient developed a focal neurological event characterized by tonic posturing of the right hemibody and clonic movements of the right upper limb lasting approximately 40 seconds, without loss of consciousness. This was followed by a generalized tonic-clonic seizure lasting five minutes, associated with loss of consciousness and sphincter control. The episode resolved after benzodiazepine administration; however, she developed right arm paresis, left leg plegia, and dysarthria. The NIH Stroke Scale (NIHSS) score was calculated as 7. Brain magnetic resonance imaging (Figure 1) was performed to rule out acute cerebrovascular disease and revealed cortical diffusion restriction on diffusion-weighted imaging (DWI) sequences. Antiepileptic treatment with levetiracetam was initiated.

She continued to have involuntary movements of the lower limbs. As part of the evaluation for FUO, cerebrospinal fluid (CSF) analysis and culture were performed, yielding negative results for bacterial or fungal infection; no pathogens grew in conventional culture media. Cytological examination of CSF showed abundant mature lymphocytes. GeneXpert sequencing for mycobacteria revealed a negative result, with no evidence of neoplastic disease. Based on MRI and CSF findings, a diagnosis of aseptic encephalitis was established. Further testing for herpes viruses, including Epstein-Barr virus, varicella-zoster virus, and cytomegalovirus, was performed, including GeneXpert testing, all of which were negative.

**Figure 2:**
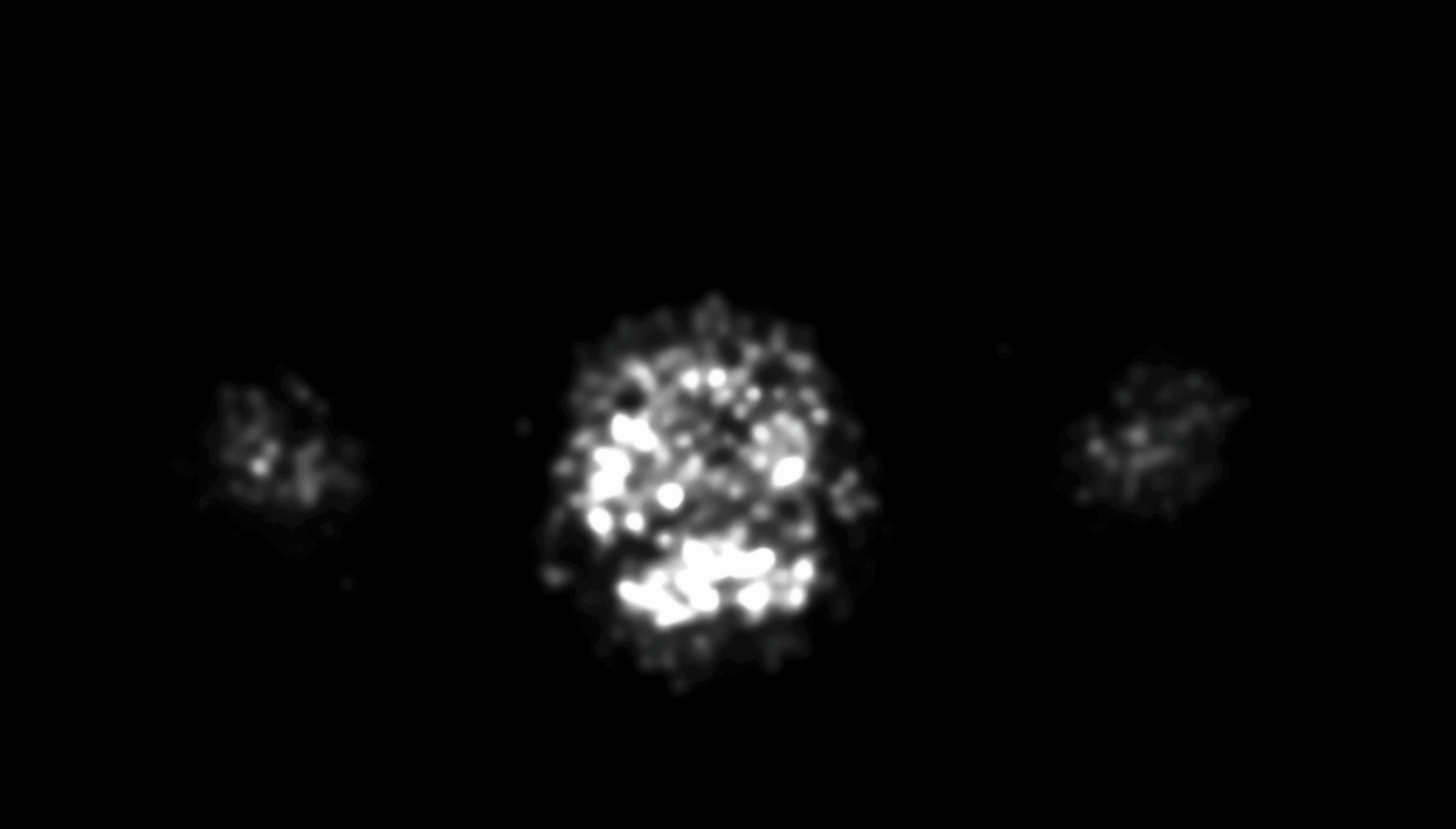
Fluorodeoxyglucose positron emission tomography/computed tomography (FDG PET/CT) imaging Clustered lymphadenopathy is noted in the left axillary region involving levels II and III.

A cervical lymph node biopsy and biopsy of the right breast mass were performed, both of which were negative for malignancy. As part of the diagnostic workup, fluorodeoxyglucose positron emission tomography/computed tomography (FDG PET/CT) showed no hypermetabolic lesions suggestive of a primary tumor (Figure 2). Hypermetabolic infra- and supradiaphragmatic lymphadenopathy was observed, which did not rule out low-grade lymphoproliferative disorders.

Pleural fluid obtained by thoracentesis was exudative according to Light’s criteria [10]. Cultures, molecular testing for Mycobacterium tuberculosis, GeneXpert assay for 32 serotypes, and histopathological examination were all negative for infection and malignancy. To further exclude lymphoproliferative disorders, myeloculture was performed and was negative. Bone marrow aspiration and biopsy revealed reactive bone marrow only. Immunohistochemistry showed CD61 positivity in megakaryocytes; CD117, CD34, CD20, and CD138 were negative; and CD68 was positive in some myeloid precursors. Due to loss of the myeloid-to-erythroid ratio, BCR/ABL1 testing was performed and was negative.

In parallel with the infectious and lymphoproliferative workup, autoimmune inflammatory diseases were investigated according to the patient’s age group. Anti-cyclic citrullinated peptide antibodies, rheumatoid factor, complement levels (C3 and C4), anti-SSA, anti-SSB, anti-RNP, anti-double-stranded DNA, anti-Smith, anti-Scl-70, anti-Jo-1, anti-ANCA (MPO and PR3), anti-actin, and antimitochondrial antibodies were all within normal ranges (Table 1).

**Table 1:** Antibody determination Ac anti-RNP: anti-ribonucleoprotein antibodies, Ac DNA: anti-double-stranded DNA antibodies, Ac SM: anti-Smith antibodies, C3: complement component 3, C4: complement component 4, anti-ANCAc PR3: cytoplasmic antineutrophil cytoplasmic antibodies (proteinase 3), anti-ANCAp MPO: perinuclear antineutrophil cytoplasmic antibodies (myeloperoxidase), anti-SCL-70: anti-topoisomerase I antibodies, anti-JO-1: anti-histididyl-tRNA synthetase antibodies, anti-mitochondrial: anti-mitochondrial antibodies, anti-actin (ASMA): anti-smooth muscle antibodies, anti-SSA (Ro): anti-Sjögren's syndrome-related antigen A, anti-SSB (La): anti-Sjögren's syndrome-related antigen B The minus sign indicates a negative result.

Immunological profile					
Date	23.05.2024	27.05.2024	06.06.2024	07.06.2024	28.06.2024
Ac Anti-RNP (UI/ mL)		6.4 (-)	3.73 (-)		2.16 (-)
Ac DNA (UI/mL)	71 (-)	46.2 (-)	45.79 (-)		3.45 (-)
Ac SM (UI/mL )	3.6 (-)	4.9 (-)	3.98 (-)		21.03 (-)
Citrulline C-peptide (UI/mL)		4.6 (-)			
Rheumatoid factor (UI/mL)		14.9 (-)			
C3 (mg/dL)	173 (normal)	121 (normal)	160 (normal)		127 (normal)
C4 (mg/dL)	25.2 (normal)	26.7 (normal)	32.4 (normal)		31.1 (normal)
Anti-ANCAc PR3 (UI/mL)			4.22 (-)		3.17 (-)
Anti-ANCAp MPO (UI/mL)			2.88 (-)		3.16 (-)
Anti-SCL-70 (UI/mL)			4.6 (-)		2.88 (-)
Anti-JO-1 (UI/mL)					3.82 (-)
Anti-mitochondrial (UI/mL)			3.96 (-)		
Anti-actin (UI/mL)			18.34 (-)		
Anti-SSA (UI/mL)			118.2 (-)		98.43 (-)
Anti-SSB (UI/mL)			1.8 (-)		1.68 (-)

Laboratory evaluation also revealed elevated IgG levels with albumin/globulin dissociation in serum protein electrophoresis, and immunofixation demonstrated a polyclonal IgG pattern, partially supporting an inflammatory/autoimmune process.

Given negative microbiological and histopathological studies, along with negative autoimmune testing, neoplastic, infectious, and autoimmune etiologies were ruled out. Initial treatment with systemic corticosteroids and colchicine led to improvement, with resolution of fever and myoclonus. Due to high suspicion of adult-onset Still’s disease complicated by aseptic encephalitis, treatment with tocilizumab at a dose of 8 mg/kg administered over one hour as a single dose was initiated. The patient showed marked clinical improvement, with resolution of fever, diaphoresis, and myoclonus. She continues treatment with tocilizumab and has had no recurrence of symptoms to date. The patient currently remains on anti-seizure medications for the prevention of further epileptic seizures.

## Discussion

Adult-onset Still’s disease is a rare idiopathic inflammatory disorder with an estimated incidence ranging from 0.16 to 0.4 cases per 100,000 individuals, depending on the population studied [8,9]. Although it predominantly affects young adults between 16 and 35 years of age, with a slight female predominance, an increasing number of cases have been reported in older populations [8,11].

According to Kong et al., spiking fevers are a universal finding in AOSD patients. While the characteristic maculopapular rash and polyarthralgia are observed in the vast majority of cases--95% and 90%, respectively--atypical presentations like the one described here remain a diagnostic challenge. In their study, additional manifestations such as lymphadenopathy, hepatosplenomegaly, and liver dysfunction were observed in 66%, 57%, and 62% of patients, respectively. Laboratory abnormalities were nearly universal, including hyperferritinemia (99%), elevated erythrocyte sedimentation rate (96%), and neutrophilic leukocytosis (98%) [7]. Autoimmune markers such as antinuclear antibodies and rheumatoid factor were notably absent in the majority of patients.

Despite her age, the patient presented with evening-predominant fever, diaphoresis, aseptic encephalitis, arthralgia, serositis, lymphadenopathy, splenomegaly, leukocytosis with neutrophilia, elevated acute-phase reactants, particularly C-reactive protein, and hyperferritinemia (exceeding 1,000 ng/mL), fulfilling three major and two minor Yamaguchi criteria. Adult-onset Still’s disease is a diagnosis of exclusion; therefore, infectious, neoplastic, and autoimmune diseases such as systemic lupus erythematosus and rheumatoid arthritis must be thoroughly ruled out.

The characteristic evanescent rash was notably absent in this patient. Although present in up to 75% of cases, its absence remains uncommon. Neurological involvement, including encephalitis, should be considered within the spectrum of AOSD complications. While rare compared with other systemic manifestations, its presence in this patient adds clinical significance and uniqueness to the case.

The patient demonstrated an initial favorable response to systemic corticosteroids; however, the most significant clinical improvement occurred following initiation of tocilizumab at a dose of 8 mg/kg administered over one hour as a single dose.

The pathophysiology of AOSD involves dysregulated immune activation, with proinflammatory interleukins playing a central role. Interleukin-1 is a key mediator, correlating strongly with disease activity and severity [3,12-14]. Interleukin-18 also contributes significantly to hyperferritinemia and systemic inflammation, with a strong association with neutrophil activation [3,15].

The Yamaguchi criteria (Table 2) remain the most sensitive diagnostic tool, with a reported sensitivity of 93.5%-96.2% and specificity of 92.1% [6,16,17]. Despite the importance of cutaneous findings, rash absence should not delay diagnosis when other criteria are fulfilled [18,19].

**Table 2:** Fulfillment of Yamaguchi criteria A diagnosis of adult-onset Still's disease (AOSD) requires at least five criteria, including two or more major criteria. This patient fulfilled three major and two minor criteria. RF: rheumatoid factor, ANA: antinuclear antibodies Adapted from [17].

Classification	Yamaguchi criterion	Presence in the patient
Major criteria	Fever ≥ 39°C (≥1 week)	Yes
	Arthralgia/Arthritis (≥2 weeks)	Yes
	Typical evanescent rash (salmon-pink)	No
	Leukocytosis (≥10,000/mm3, >80% neutrophils)	Yes
Minor criteria	Sore throat	Yes
	Lymphadenopathy and/or splenomegaly	Yes
	Liver dysfunction (elevated transaminases)	No
	Negative RF and ANA	Yes

Recent European Alliance of Associations for Rheumatology (EULAR) and the Paediatric Rheumatology European Society (PReS) 2024 recommendations classify AOSD and systemic juvenile idiopathic arthritis as a single disease continuum termed Still’s disease, emphasizing early biologic therapy to alter disease progression [20]. Early use of IL-1 or IL-6 inhibitors is recommended, reserving high-dose glucocorticoids for severe or life-threatening complications and preventing steroid therapy side effects [20,21].

## Conclusions

This case highlights the importance of considering adult-onset Still’s disease in the differential diagnosis of fever of unknown origin, even in the absence of the classic evanescent rash. Recognition of atypical presentations and keeping them in the differential diagnosis are essential to improve diagnostic accuracy and prevent the common pitfall of diagnostic delay. Neurological complications associated with AOSD are rare, with aseptic meningitis being the most frequent manifestation. Strict application of validated criteria, such as the Yamaguchi criteria, facilitates early diagnosis and timely initiation of targeted therapy, which is critical for controlling systemic inflammation and preventing life-threatening complications like macrophage activation syndrome. Furthermore, this case emphasizes the necessity of a thorough exclusionary workup to rule out infectious, neoplastic, and other autoimmune etiologies that mimic this condition. By maintaining a high clinical suspicion in patients with persistent hyperferritinemia and polyarthritis, clinicians can optimize management strategies. The initiation of early and aggressive systemic treatment not only seeks to control fever and arthritis but is also fundamental to halting the cytokine cascade before it progresses to irreversible organ damage. Prioritizing immediate systemic intervention when atypical phenotypes are suspected constitutes the most effective diagnostic and therapeutic measure to safeguard neurological integrity and ensure patient survival. Ultimately, a multidisciplinary approach and prompt intervention are key factors in achieving rapid remission, minimizing steroid dependence, and significantly improving the patient's long-term quality of life and functional outcomes.
